# The final assessment and its association with field assessment in patients who were transported by the emergency medical service

**DOI:** 10.1186/s13049-018-0579-x

**Published:** 2018-12-27

**Authors:** Carl Magnusson, Christer Axelsson, Lena Nilsson, Anneli Strömsöe, Monica Munters, Johan Herlitz, Magnus Andersson Hagiwara

**Affiliations:** 1 Department of Molecular and Clinical Medicine, University of Gothenburg and Sahlgrenska University Hospital, SE-405 30 Gothenburg, Sweden; 2 Centre for Prehospital Research, Faculty of Caring Science, Work Life and Social Welfare, University of Borås, SE-501 90 Borås, Sweden; 3 Department of Anaesthesiology and Intensive Care and Department of Medical and Health Sciences, Linköping University, SE-581 85 Linköping, Sweden; 4 School of Education, Health and Social Studies, Dalarna University Falun, SE-791 88 Falun, Sweden; 5 Department of Ambulance Care, Region of Dalarna, SE-791 29 Falun, Sweden; 6 Department of Prehospital Care, County Council of Dalarna, S-79129 Falun, Sweden

**Keywords:** Prehospital, Assessment, Diagnose

## Abstract

**Background:**

In patients who call for the emergency medical service (EMS), there is a knowledge gap with regard to the final assessment after arriving at hospital and its association with field assessment.

**Aim:**

In a representative population of patients who call for the EMS, to describe a) the final assessment at hospital discharge and b) the association between the assessment in the field and the assessment at hospital discharge.

**Methods:**

Thirty randomly selected patients reached by a dispatched ambulance each month between 1 Jan and 31 Dec 2016 in one urban, one rural and one mixed ambulance organisation in Sweden took part in the study. The exclusion criteria were age < 18 years, dead on arrival, transport between health-care facilities and secondary missions. Each patient received a unique code based on the ICD code at hospital discharge and field assessment.

**Results:**

In all, 1080 patients took part in the study, of which 1076 (99.6%) had a field assessment code. A total of 894 patients (83%) were brought to a hospital and an ICD code (ICD-10-SE) was available in 814 patients (91% of these cases and 76% of all cases included in the study). According to these ICD codes, the most frequent conditions were infection (15%), trauma (15%) and vascular disease (9%). The most frequent body localisation of the condition was the thorax (24%), head (16%) and abdomen (13%). In 118 patients (14% of all ICD codes), the condition according to the ICD code was judged as time critical. Among these cases, field assessment was assessed as potentially appropriate in 75% and potentially inappropriate in 12%.

**Conclusion:**

Among patients reached by ambulance in Sweden, 83% were transported to hospital and, among them, 14% had a time-critical condition. In these cases, the majority were assessed in the field as potentially appropriate, but 12% had a potentially inappropriate field assessment. The consequences of these findings need to be further explored.

**Electronic supplementary material:**

The online version of this article (10.1186/s13049-018-0579-x) contains supplementary material, which is available to authorized users.

## Introduction

A large proportion of patients with acute symptoms and a variety of diseases are seen by health-care providers in the emergency medical service (EMS) system [1]. These patients have a wide spectrum of diseases and the degree of urgency in terms of further evaluation and treatment varies considerably.

Previous studies [2–4] of prehospital patient safety have speculated that the main threat to prehospital patient safety is bias in clinical reasoning and decision-making. One consequence of this bias could be diagnostic errors which can lead to EMS clinicians failing to treat the patient as specified in prehospital guidelines [5]. Another risk is diagnostic momentum, which is described as a diagnostic error following the patient through the chain of care [6].

There is a lack of knowledge about the final outcome for the majority of EMS missions, as well as a shortage of knowledge of EMS clinicians’ diagnostic abilities. There is one study [7] which has described emergency physicians’ diagnostic ability in prehospital care. There are also some studies which have evaluated EMS clinicians’ diagnostic accuracy in connection with specific diagnoses, such as stroke [8] and sepsis [9, 10]. The epidemiology of EMS clinicians’ work is mostly described according to findings at the scene or, at best, according to findings and assessments on arrival at the emergency department (ED) [8, 9].

Few studies have described the epidemiology of EMS missions according to the final hospital assessment [11]. However, a final assessment can only be made in patients who have been transported by the EMS to a health-care unit, mostly the ED at a hospital.

Furthermore, not much is known about the appropriateness of the field assessment as made by an EMS clinician at the scene and how an assessment of this kind relates to the final assessment a few days later in patients who are brought to a hospital. Even less is known about the appropriateness of the field assessment in patients for whom a decision was made to let them stay on the scene with advice for self-care.

In order to understand more about the appropriateness of the field assessments that were made by EMS clinicians, there is an urgent need to study the association between field assessment and the final hospital assessment.

There are some problems when attempting to compare prehospital assessments with the final assessment at a hospital. One reason is the number of differential diagnoses related to a single initial symptom. For example, the symptom of “chest discomfort” relates to a number of differential diagnoses. Another problem is that EMS clinicians sometimes describe the patient in terms of symptoms and sometimes in terms of diagnoses. It is therefore often problematic to evaluate the clinical consequences of a prehospital assessment.

Against this background, the present study has the following aims:

1. To describe the epidemiology of the EMS clinicians’ work in relation to the final assessment after arrival at hospital
2. To describe the association between the field assessment and the final hospital assessment with the aim of critically evaluating the appropriateness of the field assessment. The final goal of an approach like this was to highlight the eventual need for an improvement in the early decision-making by EMS clinicians.
3. To relate the final assessment to the estimated risk at the scene with particular emphasis on the patients who had a final assessment equivalent to a time-critical condition.

## Methods

### Design

The study had a retrospective observational design where 30 prehospital medical records were chosen at random for screening each month in three prehospital organisations in Sweden during 1 year.

### Materials

The study material consisted of prehospital and hospital medical records. The 30 records were randomly selected by a random number generator. The 30 records were first screened for inclusion and exclusion criteria. In cases of excluded records, the procedure was repeated until 30 records which fulfilled the inclusion criteria and had no exclusion criteria were sampled. A total of 1080 ambulance missions were included in the study. Originally, there were 1664 patients who fulfilled the inclusion criteria and 584 of them were excluded.

Inclusion criterion: An ambulance mission which included patient assessment and care.

Exclusion criteria: 1) Patient aged < 18 years, 2) Ambulance missions without patient contact, 3) Ambulance missions which were secondary support to another ambulance team, 4) Ambulance transportation between health-care facilities, 5) Patient deceased upon EMS arrival.

The study excluded children, as there are specific conditions for field assessments among children with other criteria for field assessment [12].

### Setting

The study was conducted in three prehospital organisations in Sweden during 1 year, starting on 1 January 2016 and ending on 31 December 2016. The organisations were chosen so that they represented three different geographical areas in Sweden. One organisation represented an urban area with generally short transport times. The second organisation represented a mixed area with a combination of urban and rural areas including both long and short transport times. The third organisation represented mostly rural areas with the majority of cases having long transport times. We estimate that this study population gives a reasonable representation of prehospital care in Sweden.

The ambulance clinicians in the three participating organisations included registered nurses (RN), with and without a specialist education in prehospital care, and emergency medical technicians (EMT), with a shorter medical education. One ambulance team could thus consist of either two RNs or one RN and one EMT. It was always the RN that had the medical responsibility in the team. Since 2005, all ambulances in Sweden have been obliged to be run by at least one RN [13].

In each participating organisation, there was one primary reviewer. These reviewers were all active ambulance nurses working in the organisation. Secondary reviewers consisted of two researchers/ambulance nurses and two researchers/medical doctors, one cardiologist and one anaesthesiologist. The primary reviewer made the first decision on the association between the field assessment and the final hospital assessment. In cases where there was any doubt about the association, a medical doctor in the review team reviewed the case and made the final decision.

### Instruments used

**Fig. 1:**
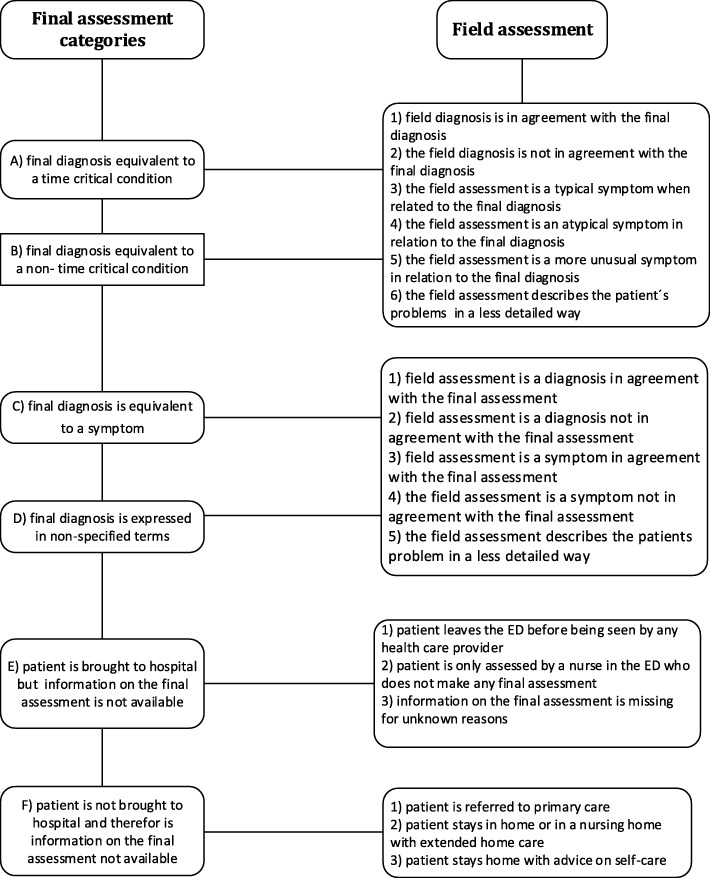
Comparisons between the field assessment and the final hospital assessment

The instruments that were used were designed to compare the field assessment with the final hospital Sassessment (Fig. 1). The patients were categorised in one of six major categories according to the final diagnosis and depending on: a) the seriousness of the diagnosis (time-critical condition or not); b) the precision of the assessment (reflecting a disease such as myocardial infarction or a symptom such as chest pain or a more diffuse condition (such as disorientation or feeling of malaise or fatigue) and c) the availability of the final assessment (sometimes not available).

As a result, the six major categories according to the final assessment were:

A) a final diagnosis equivalent to a time-critical condition (i.e. myocardial infarction or pulmonary embolism)
B) a final diagnosis equivalent to a non-time-critical condition (i.e. herpes zoster or cystitis)
C) the final diagnosis is equivalent to a symptom (chest pain or dyspnoea)
D) the final diagnosis is expressed in non-specified terms (desorientation or feeling of malaise and fatigue).
E) the patient is brought to hospital, but information on the final assessment is not available
F) the patient is not brought to hospital and information on the final assessment is therefore not available

This means that the first category includes time-critical conditions in contrast to the following categories.

The next step is to evaluate the association between the field assessment and the final assessment. For categories A and B, there are six possibilities:

1. the field diagnosis is in agreement with the final diagnosis
2. the field diagnosis is not in agreement with the final diagnosis
3. the field assessment is a typical symptom when related to the final diagnosis (i.e. chest discomfort when the final diagnosis is a myocardial infarction)
4. the field assessment is an atypical symptom in relation to the final diagnosis (i.e. dyspnoea, when the final diagnosis is a myocardial infarction)
5. the field assessment is a more unusual symptom in relation to the final diagnosis (i.e. abdominal pain when the final diagnosis is myocardial infarction)
6. the field assessment describes the patient’s problems in a less detailed way (i.e. “problems with circulation” or “problems with the airways”).

For categories C and D, there are only five subcategories, as the final assessment is not specified as a diagnosis (Fig. 1).

These subcategories are:

1. the field assessment is a diagnosis in agreement with the final assessment
2. the field assessment is a diagnosis not in agreement with the final assessment
3. the field assessment is a symptom in agreement with the final assessment
4. the field assessment is a symptom not in agreement with the final assessment
5. the field assessment describes the patient’s problem in a less detailed way, as described in categories A and B subcategory 6.

For category E, there are three subcategories:

1. the patient leaves the ED before being seen by any health-care provider .
2. the patient is only assessed by a nurse at the ED who does not make any final assessment.
3. information on the final assessment is missing for unknown reasons.

For category F, there are three subcategories:

1. the patient is referred to primary care
2. the patient stays in home or in a nursing home with extended home care
3. the patient stays at home with advice on self-care.

The final hospital assessment was related not only to the field assessment in terms of diagnosis or symptoms at the scene but also to the risk assessment at the scene. The risk at the scene was assessed using an instrument called the Rapid Emergency Triage and Treatment System (RETTS) [14]. The RETTS assessment determines the time that is required until the patient is seen by a physician according to the five colours of red, orange, yellow, green and blue. The determination is based on the five vital parameters of consciousness, heart rate, blood pressure, respiratory rate and body temperature, in combination with the Emergency Signs and Symptoms (ESS) code which defines the main type of complaint (i.e. chest pain or dyspnoea etc.). Red is defined as life threatening and orange as potentially life threatening and these patients should be monitored and a physician assessment made as soon as possible. Yellow and green are defined as non-life threatening and can wait regarding individual medical risk and both categories should be assessed by a physician within a reasonable time. Blue is the lowest triage level and referral to other levels of care may be more appropriate.

The following diagnoses were classified as time-critical conditions: anaphylaxis, myocardial infarction, unstable angina pectoris, TIA/stroke, unconsciousness, septicaemia, aortic rupture, aortic dissection, any form of shock, pulmonary embolism, heart failure including pulmonary oedema, failing heart conducting system, cardiac arrest, intoxication, status epilepticus, obstructive airway, tension/open pneumothorax, cardiac tamponade/contusion, pulmonary contusion, massive haemothorax, flail chest, oesophageal/trachea bronchial/diaphragm rupture.

This study is part of larger prehospital patient safety project and is further described in a published study protocol [15]. The instrument used for comparing field assessments with the final hospital diagnosis is also described in another publication [11].

### Statistical analysis

For all statistical analyses and data processing, the SAS package, version 9.1, was used.

The results are presented as percentages. When women were compared with men and younger patients were compared with the elderly, Fisher’s exact test was used for dichotomous variables. A p- value of < 0.05 was regarded as significant.

### Research ethics

The study was approved by the Regional Ethics Committee, Gothenburg, Sweden (Dnr 047–15).

## Results

In all, there were 1080 patients who fulfilled the inclusion criterion and did not have any exclusion criteria. Among these patients, 1076 (99.6%) were given a field assessment code.

The remainder of this article will deal with these 1076 patients.

### Final destination

Among the 1076 patients, 894 (83.1%) were transported to hospital. In 19 cases (1.8%), the patients were transported to primary care. In 16 cases (1.5%), the patients were left at home or its equivalent with extensive home care and, in 147 cases (13.7%), the patients were left at home with advice on self-care.

### Final diagnosis

In principle, a final diagnosis was available for all the patients who were brought to hospital, with the exception of 11 patients who left the ED without being assessed by a physician, 16 patients who were only assessed by a specialist nurse at the ED before discharge and a further 53 cases for whom a final diagnosis was not available for unclear reasons. In overall terms, a final assessment was thus reported in 814 patients (76% of all available cases).

**Fig. 2:**
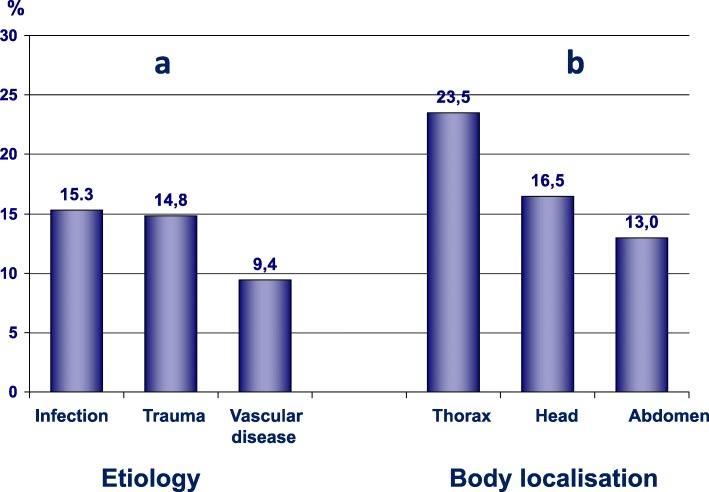
The most frequent conditions in relation to etiology (**a**) and body localisation (**b**)

The most frequent main groups of conditions according to the final diagnosis were infection, which occurred in 125 cases (15.4%), trauma in 121 cases (14.9%) and vascular diseases in 77 cases (9.5%) (Table 1, Fig. 2).

**Table 1:** The most frequent conditions (*N* = 816)

	Number	Percent
Infection	125	15.3
Trauma	121	14.8
Vascular disease	77	9.4
Psychiatric diseases	60	7.4
Neurology (stroke excluded)	53	6.5
Arrhythmia	31	3.8
Cancer	18	2.2
Degeneration in back, pelvis	17	2.1
Dyspné and respiratory insufficiency	16	2.0
Heart failure	11	1.3

The most frequent body localisation of the condition was the thorax in 192 cases (23.5%), head in 135 cases (16.5%), abdomen in 106 cases (13.0%) and lower extremities in 60 cases (7.4%).

In Table 2 is shown the distribution of patients according to the classification into the 22 major groups of ICD codes. In the Additional file 1: Table S1 is the same table expanded given the number of patients with each separate ICD code within each major ICD classification.

**Table 2:** Distribution of patients in the 22 major groups of ICD-codes (ICD-10-SE)

I	Certain infections and parasites diseases	A00 – B99	*N* = 27
II	Neoplasms	C00 – D48	*N* = 18
III	Diseases of the blood and blood forming organs and certain diseases involving the immune mechanisms	D50 – D89	*N* = 6
IV	Endocrine, nutritional and metabolic diseases	E00 – E90	*N* = 8
V	Mental and behavioural disorders	F00 – F99	*N* = 47
VI	Disease of the nervous system	G00 – G99	*N* = 27
VII	Diseases of the eye and adnexa	H00 – H59	*N* = 0
VIII	Diseases of the ear and mastoid process	H60 – H95	*N* = 6
IX	Diseases of the circulatory system	I00 – I99	*N* = 112
X	Diseases of the respiratory system	J00 – J99	*N* = 71
XI	Diseases of the digestive system	K00 – K93	*N* = 49
XII	Diseases of the skin and subcutaneous tissue	L00 – L99	*N* = 3
XIII	Diseases of the musculoskeletal tissue and connective tissue	M00 – M99	*N* = 43
XIV	Diseases of the genitourinary system	N00 – N99	*N* = 37
XV	Pregnancy childbirth and puerperium	O00 – O99	*N* = 2
XVI	Certain conditions originating from the perinatal period	P00 – P96	*N* = 0
XVII	Congenital malformations, deformation and chromosomal malformations	Q00 – Q99	*N* = 0
XVIII	Symptoms, signs and abnormal clinical findings, not elsewhere classified	R00 – R99	*N* = 188
XIX	Injury, poisoning and certain other consequences of external causes	S00 – T98	*N* = 135
XX	External causes of morbidity and mortality	V00 – V99	*N* = 0
XXI	External causes to disease and death	Y01 – Y98	*N* = 5
XXII	Factors influencing health status and contact with health services	Z00 – Z99	*N* = 30

### Field assessment in relation to final outcome

The distribution of patients according to the six main categories is shown in Table 3. In 114 patients (14.0% of all cases in which a final diagnosis was available), the condition was categorised as a time-critical condition according to final outcome (ICD code). In 87 patients (75.0% of the 114 cases), the field assessment was judged as potentially appropriate (A1 or A3). In 14 cases (12.0%), the field assessment was judged as potentially inappropriate (A2 or A6). The final diagnoses among the 14 patients who were judged as potentially inappropriate are shown in Table 4.

**Table 3:** Association between the field assessment and the final diagnosis

	A^a^	B^b^	C^c^	D^d^
Field assessment	*N* = 114	*N* = 439	*N* = 185	*N* = 71
1. The field diagnosis is in agreement with the final diagnosis	47(41)^e^	102(23)	16(9)	11(15)
2. The field diagnosis is not in agreement with the final diagnosis	9(8)	58(13)	10(5)	12(17)
3. The field assessment is a typical symptom when related to the final diagnosis	40(34)	167(13)	142(77)	133(46)
4. The field assessment is not a typical symptom when related to the final diagnosis	15(13)	39(9)	12(6)	6(8)
a/ atypical symptoms	13(11)	30(7)	–	–
b/ unusual symptoms	2(2)	9(2)	–	–
6. The field assessment is less detailed	5(4)	73(17)	5(3)	9(13)

^a^= A final diagnosis equivalent to a time critical condition

^b^= A final diagnosis equivalent to a non time critical condition

^c^= The final diagnosis is equivalent to a symptom

^d^= The final diagnosis is expressed in non specified terms

^e^*n* = (%)

**Table 4:** Final diagnoses among patients in whom field assessment was judged as potentially inappropriate (*n* = 14). Missing = 1

	N
Sepsis	3
Myocardial infarction	2
Cerebral infarction	2
Subdural bleeding	2
Cerebellar bleeding	1
Subarachnoidal bleeding	1
Aorta dissection	1
Status epilepticus	1

In 439 patients (53.9%), the condition was categorised as a specified non-time-critical condition according to final outcome (ICD code). In 269 of these cases (61.2%), the field assessment was judged as potentially appropriate (B1 or B3). In 129 patients (29.8%), the field assessment was judged as potentially inappropriate (B2 or B6).

In 185 patients (22.7%), the final outcome according to the ICD code was described as a symptom (Category C) and, in 70 patients (8.6%), the final outcome was described in more general terms according to the ICD code (Category D).

Among patients who were classified as Category A (*n* = 114), the patients were distributed according to RETTS colour, as shown in Table 5. As a result, 39 (34%) were given a red colour (the highest priority) and 86 patients (75%) were given one of the two highest priorities. There was no significant difference when the distribution was related to age or gender (Table 5).

**Table 5:** Distribution of patients according to initial priority among patients with a time critical condition (A)

	All patients	Women	Men	p	Age ≤ median^a^	Age > median	p
Retts colours	*N* = 114	*N* = 43	*N* = 71		*N* = 56^b^	*N* = 56	
				0.78			0.30
Red	39(34)^c^	13(30)	26(37)		20(36)	17(30)	
Orange	47(41)	20(47)	27(38)		25(45)	22(39)	
Yellow	24(21	9(21)	15(21)		9(16)	15(27)	
Green	4/4)	1(2)	3(4)		2(4)	2(4)	

^a^ Median age = 74.5 years

^b^Two cases missing

^c^*n* = (%)

### Patients who are not transported to hospital

Among these patients (*n* = 182), 26 (14.6%; missing information in four patients) sought medical care ≤72 h after the first assessment and nine (5.1%; missing information in four patients) died ≤30 days after the first assessment.

## Discussion

In this study comprising 1080 patients, who were representative of patients seen by an EMS clinician, the main findings were as follows.

The most frequent group of conditions according to the final ICD code was infection, followed by trauma, followed by a vascular disease. In 14% of the cases in which an ICD code was available, the condition was classified as time critical. Among these cases, the field assessment was judged to be potentially inappropriate in 12% of the cases.

The observation that patients with an infection constituted the most frequent group of patients is new, but it must also be related to the fact that many of these infections were relatively harmless, did not require hospitalisation and only a minority (2%) had a severe condition like sepsis.

To the best of our knowledge, the observation that trauma represents the second largest group of patients seen by the EMS among those with available ICD codes has not previously been reported. Here, it is important to highlight that all types of trauma were included, many of which were minor and not always requiring hospitalisation.

The group of patients who had a confirmed vascular disease as the underlying aetiology could be regarded as fewer than expected (only 9%). This should be related to the fact that chest pain and dyspnoea are the most frequent symptoms in an ambulance-transported patient cohort [16]. However, only a minority of these patients have a final diagnosis indicating vascular disease aetiology such as myocardial infarction. This is in agreement with previous findings [17].

We found that only 14% of patients with an available ICD code had a time-critical condition according to the given criteria. In a recently published study of patients who used the EMS on multiple occasions, the corresponding figure was 13% [11]. We found that, among these patients, the field assessment could be retrospectively judged to be potentially inappropriate in 12%. The corresponding figure in the study previously referred to was 22% [11]. These cases can be regarded as representing the greatest risks of an adverse event. Among patients with a time-critical condition and a potentially inappropriate field assessment, the final diagnoses most frequently represented patients with cerebrovascular disease, followed in order of frequency by sepsis, myocardial infarction, aortic dissection and status epilepticus.

Among the patients with a time-critical condition, only 34% were given the highest priority by the EMS nurse. This finding highlights the need for better instruments to improve the initial prioritisation in the future. Our results also highlight the fact that the field assessment and initial priority may not always provide the same information.

Nurses have always been involved in the diagnostic process [18] and, for EMS clinicians, establishing a field diagnosis is an important task [19]. A correct field diagnosis is important for several reasons. On many occasions, the treatment in the prehospital setting is based on diagnoses and, with an incorrect assessment there is a risk that the patient will not receive the treatment which has been specified in the prehospital guidelines. In many cases, some specific conditions are indications for direct transport to facilities for specialist assessment and treatment, thereby bypassing the ED. Examples of this are patients with an ST-elevation myocardial infarction [20], stroke [21] and sepsis [22]. Another problem with prehospital diagnostic errors is that important information in the hand-over and in medical journals may be missing.

Diagnostic error is a major patient safety threat in health care, but it is difficult to estimate diagnostic error rates [23, 24]. Emergency medicine is an area in which diagnostic errors are a common reason for adverse events [25]. Kachalia et al. [26] listed several reasons for diagnostic errors at the ED. The most common factor was cognitive factors, such as knowledge and judgement, followed by system factors, such as workload and interruptions, communication factors, such as hand-overs and conflicts, and patient-related factors, such as patient non-adherence and a complicated medical history. Croskerry [27] highlights cognitive bias as the most important factor in relation to diagnostic errors and a systematic review confirms this view [28]. The reasons for cognitive bias can be explained by the dual process theory [29] in which human reasoning is divided into two systems. System 1 is described as a subconscious, automatic, rapid system. System 1 is dependent on experience and the most common bias is premature closure (omission bias) where the decision-maker makes decisions too early in the process. System 2 is described as an analytical, slow process and is the most commonly used system in new and complex situations. The most common errors in System 2 processes are mistakes of commission where the clinicians gather too much information and thereby risk cognitive overload [29, 30]. Cognitive processes are sparsely investigated in prehospital care. The few studies reveal a similarity in the cognitive methods used in the prehospital setting compared with methods used in in-hospital emergency care [31–33]. It has been suggested that the main difference between in-hospital and prehospital emergency care is the settings in which clinical reasoning is executed [34]. Another feature suggested to differentiate prehospital clinical reasoning from the in-hospital setting is the frequent use of cognitive tools such as guidelines and protocols [34]. This form of reasoning has been labelled System 2-by proxy. It includes elements of algorithmic reasoning and ruling out the worst-case scenario [32]. However, there are indications that cognitive tools in the prehospital setting are frequently poorly adapted to the context [35].

In a review, Graber et al. [36] found three main interventions that reduce diagnostic errors. The first was interventions to increase knowledge and experience and the main method in this category is the use of medical simulation. The second category is interventions to improve clinical reasoning with methods like reflective practice and metacognition and the third category is cognitive tools such as decision support. Graber et al. [36] also concluded that the evidence base for these interventions was low and that there was a need for further research. Croskerry et al. suggest similar methods to prevent diagnostic errors [37]. Ely et al. [38] argue in favour of the development of checklists to prevent diagnostic errors.

The same interventions are probably applicable in prehospital care. Medical simulation has the potential to improve EMS clinicians’ clinical performance [39]. Since the use of different cognitive tools is important in prehospital care, the development of these tools is urgent. Research in the area is limited [40], but there are indications that compliance with guidelines can be increased by using a computerised decision-support system in the prehospital setting [5, 41]. However, the effect on diagnostic accuracy is unknown.

## Limitation

Our study only includes a subset of patients from three different regions in Sweden. It is therefore possible to question whether these patients should be regarded as representative of Sweden.

This was a retrospective observational study with a number of potential weaknesses. Missing data is probably the most important one. The assessment that was made by the EMS clinician can only be evaluated from what was written in the case records. In some cases, he or she may have had other thoughts that were not appropriately recorded.

Furthermore, is the frequency of various conditions based on the number of patients who had an available ICD code (76% of all patients). The true percentage values will therefore most probably be lower in a number of respects that were addressed in this manuscript.

In prehospital care, there is a knowledge gap regarding the association between the field assessment and the final assessment after arriving at hospital. Critical voices have been raised saying that these comparisons are difficult to make, as new events may have developed between the two assessments. To reduce this error, comparisons have been made with the first required diagnosis in the hospital; for example, the intoxicated patient who was given depression as a final diagnosis was not compared, but a first diagnosis of acute intoxication was made when he/she was released from the medical ward and transferred to a psychiatric ward. Therefore, it is our belief that this comparison provides valuable knowledge to prehospital patient safety research.

## Conclusions

Among patients reached by ambulance in Sweden, 83% were transported to hospital and, of them, 14% had a time-critical condition. Among these cases, the majority were assessed in the field as potentially appropriate, but 12% had a potentially inappropriate field assessment. The consequences of these findings need to be further explored.

## Additional file

Additional file 1: List of ICD-codes and the number of patients with different ICD-codes within parenthesis (ICD-10-SE) (DOCX 16 kb)

## Abbreviations

AE: Adverse event/s
ED: Emergency department
EMS: Emergency medical systems
EMT: Emergency medical technicians
RN: Registered nurses
