# Supplementary material for: The final assessment and its association with field assessment in patients who were transported by the emergency medical service
Source: Scand J Trauma Resusc Emerg Med. 2018 Dec 27;26:111. doi: 10.1186/s13049-018-0579-x (PMC6307253; doi:10.1186/s13049-018-0579-x)
Supplement: Supplementary file 1 — List of ICD-codes and the number of patients with different ICD-codes within parenthesis (ICD-10-SE) (DOCX 16 kb) [file 13049_2018_579_MOESM1_ESM.docx]

Supplementary Table 1. List of ICD-codes and the number of patients with different ICD-codes within parenthesis (ICD-10-SE)

**I Certain infections and parasites diseases:** A 40.3 (1), A 02.0 (1), A 04.7 (2), A 08.0 (1), A 08.1 (1), A 09.0 (1), A 09.9 (4), A 41.8 (1), A 41.9 (3), A 46.9 (3), A 49.9 (1), A 87.9 (1), B 33.8 (1), B 34.9 (4), B 99.9 (2)

**II Neoplasms:** C 18.1 (1), C 22.0 (1), C 25.9 (1), C 48.2 (1), C 56.9 (1), C 61.9 (2), C 67.9 (1), C 70.0 (1), C 71.3 (1), C 79.3 (2), C 81.9 (1), C 93.1 (1), D 38.1 (1), D 41.0 (1), D 42.0 (1), D 47.1 (1)

**III Diseases of the blood and blood forming organs and certain diseases involving the immune mechanisms:** D 50.9 (1), D 64.9 (4), D 74.9C (1)

**IV Endocrine, nutritional and metabolic diseases:** E 10.1A (1), E 10.8 (1), E 11.0C (1), E 16.1 (1), E 86.9 (1), E 87.0B (1), E 87.1B (1)

**V Mental and behavioural disorders**: F 02.8 (1), F 03.9 (1), F 05.9 (3), F 10.0 (4), F 10.1 (1), F 10.2 (4), F 10.2A (1), F 10.3 (1), F 10.9 (1), F 11.3 (1), F 13.0 (5), F 15.0 (1), F 19.0 (2), F 19.1 (1), F 19.2 (2), F 23.8 (1), F 32.2 (1), F 41.0 (4), F 41.2 (2), F 41.9 (5), F 43.0 (1), F 44.9 (1), F 50.8 (1), F 90.0B (1), F 99.9 (1)

**VI Disease of the nervous system:** G 03.0 (1), G 03.9 (1), G 20.9 (4), G 23.1 (1), G 23.3 (1), G 35.9 (1), G 40.4X (1), G 40.9 (2), G 41.9 (1), G 43.9 (1), G 44.2 (1), G 45.1 (2), G 45.4 (2), G 45.9 (6), G 83.8 (1), G 93.2 (1)

**VII Diseases of the eye and adnexa: ---**

**VIII**  **Diseases of the ear and mastoid process:** H 81.1 (3), H 81.2 (2), H81.3 (1)

**IX Diseases of the circulatory system:** I 10.9 (4), I 20.0 (2), I 20.1 (1), I 20.9 (3), I 21.0 (1), I 21.1 (1), I 21.3 (2), I 21.4 (6), I 21.9 (1), I 25.9 (1), I 26.9 (2), I 30.9 (1), I 33.0 (1), I 33.9 (1), I 44.1B (1), I 44.2 (2), I 46.9 (3), I 47.1 (1), I 47.2C (1), I 48.0 (5), I 48.1 (2), I 48.2 (1), I 48.9 (11), I 49.5 (1), I 50.0 (1), I 50.9 (10), I 60.9 (1), I 60.9B (1), I 61.0 (3), I 61.4 (1), I 61.9 (4), I 62.0 (1), I 63.3 (2), I 63.4 (3), I 63.5 (3), I 63.8 (1), I 63.9 (10), I 69.3 (1), I 71.0 (2), I 71.5 (1), I 74.3 (1), I 80.2 (1), I 80.3 (2), I 89.0 (1), I 95.1 (7)

**X Diseases of the respiratory system:** J 01.9 (1), J 06.9 (6), J 09.9 (1), J 10.0 (2), J 10.1 (1), J 10.8 (1), J 11.1 (1), J 15.7 (1), J 15.9 (7), J 18.9 (16), J 20.8 (1), J 32.9 (1), J 44.1 (16), J 44.9 (3), J 45.9 (1), J 69.0 (4), J 81.9 (1), J 84.1 (1), J 85.2 (1), J 90.9 (1), J 96.0 (1), J 96.1 (2), J 96.9 (1)

**XI Diseases of the digestive system:** K 22.0 (1), K 22.1 (1), K 25.0 (1), K 29.7 (4), K 30.9 (2), K 35.3 (1), 37.9 (1), K 40.3 (2), K 44.9 (1), K 45.8 (1), K 52.9 (2), K 56.5 (1), K 56.7 (3), K 57.9 (1), K 59.0 (2), K 60.3 (1), K 61.0 (1), K 62.5 (1), K 63.1 (1), K 65.0 (1), K 70.3 (1), K 70.4 (1), K 72.9 (1), K 80.2 (3), K 80.5 (1), K 80.8 (1), K 81.0 (2), K 83.0 (1), K 85.1 (1), K 85.9 (1), K 91.1 (1), K 92.1 (1), K 92.2 (5)

**XII Diseases of the skin and subcutaneous tissue:** L 02.4 (1), L 02.9 (1), L 50.0 (1)

**XIII Diseases of the musculoskeletal tissue and connective tissue:** M 00.0G (1), M 00.9F (1), M 24.4B (1), M 24.4F (1), M 25.5 (1), M 35.3 (1), M 48.5 (4), M 51.1 (1), M 51.1K (1), M 54.4 (4), M 54.5 (7), M 54.9 (5), M 79.1 (2), M 79.1C (1), M 79.6 (2), M 79.6F (5), M 79.6G (2), M 90.7 (1), M 94.0 (1), M 96.6 (1)

**XIV Diseases of the genitourinary system:** N 10.9 (6), N 17.9 (1), N 19.0 (1), N 20.0 (3), N 20.1 (3), N 20.9 (3), N 30.0 (3), N 30.1 (1), N 30.9 (2), N 39.0 (6), N 39.0X (3), N 71.0 (1), N 71.9 (1), N 80.3 (1), N 94.6 (1), N 95.0A (1)

**XV Pregnancy childbirth and puerperium:** O 02.1 (1), O 04.9 (1)

**XVI Certain conditions originating from the perinatal period:** ---

**XVII Congenital malformations, deformation and chromosomal malformations:** ---

**XVIII Symptoms, signs and abnormal clinical findings, not elsewhere classified:** R 00.2 (3), R 04.0 (8), R 04.1 (1), R 05.9 (1), R 06.0 (12), R 07.2 (1), R 07.4 (43), R 10.0 (1), R 10.1 (1), R 10.4 (5), R 10.4X (16), R 11.9 (4), R 11.9A (1), R 17.9 (2), R 20.2 (4), R 22.4 (1), R 25.2 (2), R 29.6 (1), R 31.9 (3), R 33.9 (3), R 40.2 (1), R 41.0 (2), R 42.9 (24), R 44.3 (1), R 47.0 (1), R 50.9 (1), R 51.9 (2), R 52.0 (2), R 53.9 (8), R 55.9 (16), R 56.8 (5), R 56.8X (5), R 57.2 (2), R 73.9 (5)

**XIX Injury, poisoning and certain other consequences of external causes:** S 00.9 (9), S 01.0 (3), S 01.8 (4), S 01.9 (7), S 02.2 (1), S 03.4 (1), S 06.0 (4), S 06.5 (2), S 06.6 (2), S 10.9 (1), S 12.2 (1), S 20.2 (2), S 20.4 (1), S 21.8 (1), S 22.00 (1), S 22.3 (2), S 22.4 (1), S 22.40 (2), S 27.1 (1), S 27.2 (1), S 30.0 (2), S 31.4 (1), S 32.0 (1), S 32.5O (1), S 32.8 (1), S 36.4 (1), S 40.0 (2), S 41.1 (1), S 42.2 (3), S 42.20 (3), S 42.4O (2), S 43.0 (4), S 51.7 (1), S 51.9 (1), S 52.50 (1), S 52.60 (2), S 52.8 (1), S 52.80 (1), S 60.2 (1), S 61.1 (1), S 62.61 (1), S 70.0 (7), S 70.1 (1), S 72.0 (1), S 72.00 (7), S 72.10 (5), S 72.20 (2), S 80.0 (1), S 81.0 (1), S 81.9 (1), S 82.60 (2), S 82.80 (5), S 83.0 (1), S 83.7 (1), S 86.0 (1), S 91.3 (1), S 92.00 (1), S 93.4 (2), S 96,8 (1), T 01.9 (1), T 07.9 (1), T 14.0A (1), T 14.1A (1), T 17.2 (1), T 25.2 (1), T 40.2 (1), T 50.9 (1), T 51.0 (1), T 75.0 (1), T 75.4 (2), T 78.2B (1), T 79.3 (1), T 80.2 (1)

**XX External causes of morbidity and mortality: ---**

**XXI External causes to disease and death:** W 18.09 (1), X 61.99 (1), X 64.99 (1), Y 91.0 (2)

**XXII Factors influencing health status and contact with health services**: Z 00.0 (1), Z 01.8 (1), Z 03.3 (2) Z 03.6 (4), Z 03.8 (4), Z 03.8A (1), Z 03.8B (1), Z 03.9 (7), Z 04.1 (1), Z 04.9 (1), Z 13.6 (1), Z 45.0 (1), Z 51.8 (2), Z 60.9 (1), Z 71.1 (2)
